# Atorvastatin mucoadhesive tablets in the management of recurrent aphthous stomatitis: a randomized clinical study

**DOI:** 10.1186/s12903-023-02846-x

**Published:** 2023-05-13

**Authors:** Tahereh Molania, Jafar Akbari, Amirhossein Babaei, Anahita Lotfizadeh, Mahmood Moosazadeh, Abbas Mesgarani, Anahita Baasl, Maede Salehi

**Affiliations:** 1 Department of Oral Medicine, Dental Research Center, Mazandaran University of Medical Sciences, Sari, Iran; 2 Faculty of Dentistry, Mazandaran University of Medical Sciences, Sari, Iran; 3 Department of Pharmaceutics, Faculty of Pharmacy, Pharmaceutical Sciences Research Center, Mazandaran University of Medical Sciences, Sari, Iran; 4 Department of Pharmaceutics, Faculty of Pharmacy, Mazandaran University of Medical Sciences, Sari, Iran; 5 Student Research Committee, Faculty of Dentistry, Mazandaran University of Medical Sciences, Sari, Iran; 6 Gastrointestitional Cancer Research Center, Non-communicable Disease Institute, Mazandaran University of Medical Sciences, Sari, Iran; 7 Department of Oral Endodontics, Dental Research Center, Mazandaran University of Medical Sciences, Sari, Iran

**Keywords:** Recurrent aphthous ulcer, Atorvastatin, Mucoadhesive tablet, Treatment

## Abstract

**Background:**

Aphthous stomatitis is one of the most common oral mucosal diseases. Due to the commonness of recurrent aphthous stomatitis and considering the anti-inflammatory, analgesic, and tissue regenerative properties of atorvastatin and the lack of a study on the effect of statins on minor recurrent aphthous stomatitis, this study investigates the effect of atorvastatin mucoadhesive tablets as a topical treatment on reduction of symptoms and duration of this disease.

**Methods:**

This study is a randomized, double-blinded clinical trial. Patients were divided into two groups, atorvastatin and, placebo; each of the patients received three mucoadhesive tablets daily in the morning, noon, and night. Finally, the patients were examined on days 0 (baseline), 3, 5, and 7 to determine the diameter of the inflammatory halo. The VAS scale was used to evaluate pain intensity for up to 7 days after each meal. The data was entered into SPSS 24 software and analyzed.

**Results:**

The halo diameter did not significantly differ between the two groups on baseline (P > 0.05). However, on the study’s third, fifth, and seventh days, the difference between the two groups was remarkable, so in the atorvastatin group, the size of the lesions decreased in shorter healing time (P < 0.05). In addition, the patient’s pain intensity (VAS) also showed a significant decrease in the atorvastatin group except on the first, second, and seventh days of the study (P < 0.05).

**Conclusion:**

Atorvastatin mucoadhesive tablets effectively reduce the pain of patients with minor recurrent aphthous stomatitis and reduce the size and healing time of the lesions, so their application should be considered in treating minor recurrent aphthous stomatitis. The present study was approved by the Medical Ethics Committee of Mazandaran University of Medical Sciences with the ethics code IR.MAZUMS.REC.1400.8346. Also, this study received code IRCT20170430033722N4.

## Background

Recurrent aphthous stomatitis (RAS) is an ulcerative and painful lesion with a prevalence of 5–60%, which can interfere with an individual’s nutrition, speech, and oral hygiene due to its painful nature, which in turn can affect the patient’s quality of life [1]. The etiology of this disease is multifactorial, especially inflammatory, caused by the imbalance of the T-cell-dependent immune system. Therefore factors that affect the patient’s immune response can be influential in the occurrence of this disease, including hereditary, food allergy, deficiency of vitamins and some elements, especially vitamin B_12_ and folic acid, systemic disease, hormonal imbalance, mechanical injuries, stress, etc. [2].

Aphthous stomatitis is clinically divided into minor, major, and herpetic forms. The most common form of RAS is minor, with a prevalence of more than 80%. Clinically, minor aphthous stomatitis is in the form of shallow ulcers with a size smaller than 5 mm, single or multiple, round or oval with a necrotic base and erythematous border with a burning pain that appears 2 to 48 h before the formation of the ulcer. The lesions specifically involve the non-keratinized and moveable mucosa of the mouth, including the mucosa of the lips, cheeks, lateral and ventral surface of the tongue, soft palate, and oropharynx [1, 3, 4].

Significant treatment protocols have been introduced for this condition, but the etiology of this disease is still idiopathic, and these treatments are primarily symptomatic rather than preventive [5]. Therefore, the available treatments are provided to reduce pain, healing period, size, number, and duration of the lesions. Some of these treatments include topical treatments (dexamethasone, topical lidocaine 2%, silver nitrate tablets, etc.), therapeutic mouthwashes (0.5% minocycline, sucralfate suspension, Etc.), vitamin supplements (Omega-3), systemic medical treatments (subcutaneous injection of 3 mg enoxaparin, low dose prednisolone with levamisole) and finally laser therapy; in general, topical treatments and mouthwashes are considered the first line of treatments due to fewer side effects [4].

Statins are inhibitors of the enzyme 3-hydroxyl-3methylglutaryl coenzyme A (HMG-CoA) reductase. This enzyme catalyzes the first step of cholesterol biosynthesis, so the primary role of statins is to reduce the risk of cardiovascular problems in patients with hyperlipidemia, hypertension, and type 2 diabetes [6]. In addition to this importance, various other effects have been mentioned for this substance in recent studies, including anti-inflammatory properties and immune balancing, analgesic, antioxidant, neuronal protection, improving the structure of the vessel and endothelium, etc. [7, 8]. Furthermore, in some studies, statins’ numerous effects were used in treating various diseases and conditions. For example: using the anti-inflammatory and immune balancing properties of atorvastatin, treating inflammatory diseases, including intestinal ulcers and traumatic brain injuries has been done [7, 9]. In another study, it was used topically in treating patients with periodontitis [10]. Furthermore, because of its angiogenesis and lymphangiogenesis properties, it was used as a medicine to advance wound healing in diabetic or post-surgery wounds [11, 12].

The beneficial effects of statins are related to their anti-inflammatory properties. They reduce the release of C-reactive peptides, cytokines, chemokines, and adhesion molecules; they also affect T-cell activity modulation. Statins inhibit the migration of leukocytes by decreasing the expression of adhesion molecules. Furthermore, prevent inflammation by suppressing chemokine release and Th1-type chemokine receptors of T-cells [13]. The findings of the previous studies demonstrate that the oral or topical application of statins in long-term or short-term leads to the healing of various types of wounds [14].

The topical use of these drugs can put a higher dose of the medicine in contact with the ulcer for a more extentended period and simultaneously reduce the side effects caused by systemic drug use. Studies have shown that the topical use of this substance may cause mild adverse effects such as itching, burning, and irritation. However, it is well tolerated and highly compatible with many patients [8, 15].

Given the anti-inflammatory, analgesic, and tissue regenerative properties of atorvastatin and the lack of a study on the effect of statins on minor recurrent intraoral ulcers, we decided to evaluate the efficacy of atorvastatin mucoadhesive tablets as a topical treatment on reducing the symptoms and duration of minor recurrent aphthous stomatitis.

## Methods

This randomized, double-blinded clinical trial was approved by the Medical Ethics Committee of Mazandaran University of Medical Sciences (Moral Code: IR.MAZUMS.REC.1400.8346).

All patients received a sufficient explanation about the treatment process and possible complications; then signed a consent form before entering the study.

### Participants and inclusion criteria

According to the study conducted by Babaei et al. with the mean and standard deviation of the lesion diameter on the seventh day in the control group of 0.60 ± 0.69 and the intervention group of 1.29 ± 0.66 with a confidence level of 95%, a test power of 90%, and for the two-way test, by using the formula for comparing the two averages in the G-power software. The sample size of the current study was calculated 44 patients (22 patients in each group) [16]. Based on the inclusion criteria, 44 participants were selected in the first step using a convenient sampling method. Then; the blocking method was used to divide the samples into two groups. by using a blocking method and random allocation software, samples were randomly assigned. 11 quadruple blocks were produced with this program. Based on the randomly generated numbers, the samples were included in the study.

The patient selection was based on the inclusion criteria from those referred to Mazandaran dental school with recurrent aphthous stomatitis in the age range 20–40, reporting a history of minor aphthous ulcers in areas such as the lips and buccal mucosa. The patients were randomly divided into two groups: 22 patients in the intervention group and 22 patients in the control group. The head nurse of the dental clinic (who was not from analyzers or evaluators) registered the patients and gave the medication to participants (atorvastatin mucoadhesive tablets or placebo). This intervention took 7 days.

The inclusion criteria of this study include patients with minor recurrent stomatitis, patients with aphthous lesions in lips and buccal mucosa (due to greater access and less movement that allows mucoadhesive tablets to remain on the lesion), systemically healthy, not taking immunosuppressive drugs within the past month, not using dentures, not taking antibiotics. Also, pregnant patients, people who were not able to use mucoadhesive tablets, people with syndromes manifesting aphthous-like lesions (Behcet’s syndrome), smokers, people with mucosal skin autoimmune diseases, patients with liver failure, myopathy, and muscular disorders, patients experiencing urticaria and skin and mucosal itchiness, and those who were not able to study due to personal or social reasons, were excluded from the study [15, 16].

### Developing mucoadhesive tablets

Mucoadhesive tablets were produced in the laboratory of Mazandaran medical university (Faculty of pharmacy). 10-mg atorvastatin mucoadhesive tablets were prepared with different proportions of the medicine and bioadhesive polymers such as HPMC or CMC. Tablets were prepared by direct compression method after weighing and complete mixing of medicine, polymer, and other excipients such as Avicel as a filler, Colloidal silicon dioxide as a glidant, and Magnesium stearate as a lubricant.

In the present study, atorvastatin was manufactured by Tehran Chemical Company, Avicel was produced by Saba Chemical Company, Magnesium stearate was developed by Axin Chemical Company in Iran, and Aerosol was manufactured by Avonic Company in Germany.

### Study protocol

In this study, the evaluator and all patients in both groups were blinded. The patients were informed to visit the dental clinic during the first 24 h after the occurrence of aphthous lesions, and their first visit was regarded as the baseline (day 0). During the initial visit, patients were firstly assured about the safety of the project; then they were asked to read and sign the consent form and also complete the questionnaire containing the medical and dental history of patients. In the same session, the first mucoadhesive tablet was placed on the patient’s aphthous lesion by the examiner; notably, that the evaluator in this study was blinded and not conscious of the medicine which was given to each patient. Patients were informed to use the tablets 3 times daily in the morning, noon, and night. They were instructed how to use mucoadhesive tablets and advised to avoid eating and drinking for 30 min after usage and remove the tablet after 30 min. In the control group, the same procedure was performed with a placebo which contains all materials used in atorvastatin mucoadhesive tablets, except the primary substance, which is atorvastatin. All patients were advised not to use other anti-inflammatory medicines.

To evaluate the amount of pain and healing of the lesions, the patients were clinically examined 0 (baseline), 3, 5, and 7 days later using a metal caliper to determine the diameter of the lesions and the inflammatory area around them [16]. Patients were also instructed to rate their pain intensity based on the Visual Analogue Scale (VAS). This scale includes a 10 cm line, where zero means no pain and 10 means maximum pain. The patients identified the point that characterized their pain on this scale and used a numerical scale (e.g.; from 1 to 10) to estimate the intensity of the pain. Patients recorded VAS in the questionnaire 3 times daily after each meal because the pain maximizes after the incitement induced by eating, chewing, and food particles. Patients with a pain score of 1 and a diameter of lesions less than 1 mm were considered improved [17].

### Data analysis

After completing the data and information collection, the acquired data was entered into SPSS 24 software. Then, quantitative variables were reported using the mean ± standard deviation. A Chi-square test was used to compare gender distribution between the two groups. The normal distribution of quantitative variables was evaluated using the Kolmogorov-Smirnov test. The comparison of quantitative variables between the two groups was performed by independent T-test or Mann-Whitney test. Friedman’s test was performed to assess the changes in the erythematous aura and pain score over time, separating the intervention and control groups. The Generalized Estimated Equations (GEE) test was used to examine changes in the diameter of the inflammatory aura and pain intensity between the two groups over 7 days. A significance level of 0.05 was considered for all tests.

## Results

**Fig. 1:**
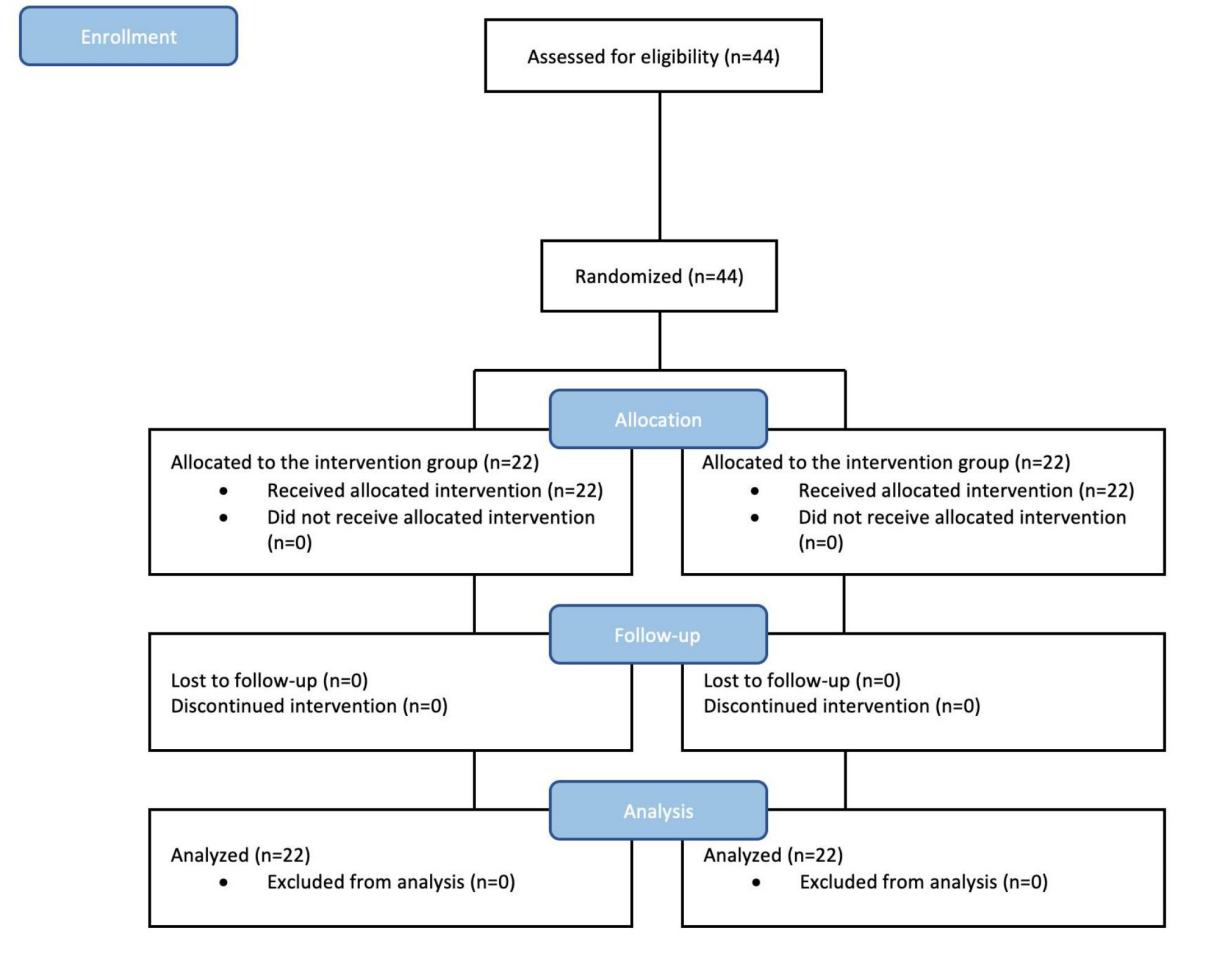
CONSORT flowchart

This study was conducted as a double-blinded clinical trial. Somehow, 44 patients who met the inclusion criteria of the study were selected from those who were referred to the dental clinic of Mazandaran University of Medical Sciences (18 men and 26 women) and completed the treatment and study period. At the same time, they were divided into two groups: intervention (Atorvastatin) and control (Placebo) groups. 22 patients were included in each study group and all patients were studied and examined for 7 days. To evaluate the diameter of the inflammatory halo, patients were clinically examined on the baseline (day zero), 3rd, 5th, and 7th days. The patients were also taught to determine the intensity of pain based on the VAS scale. Participants in this study reported no side effects or complaints from the mucoadhesive tablets (Fig. 1).

The comparison of gender in the two groups showed that the distribution of gender in control (female: 59.1%, male: 40.9%), and atorvastatin (female: 59.1%, male: 40.9%) groups were not significantly different from each other and they were homogeneously distributed (P = 0.999). Also, the mean and standard deviation of the age in control (14.27 ± 39.09) and atorvastatin (13.64 ± 34.81) groups were examined, and according to the results of the Mann-Whitney test, the mean age in the two groups did not show a statistically significant difference (P = 0.372).

### Inflammatory halo diameter

**Fig. 2:**
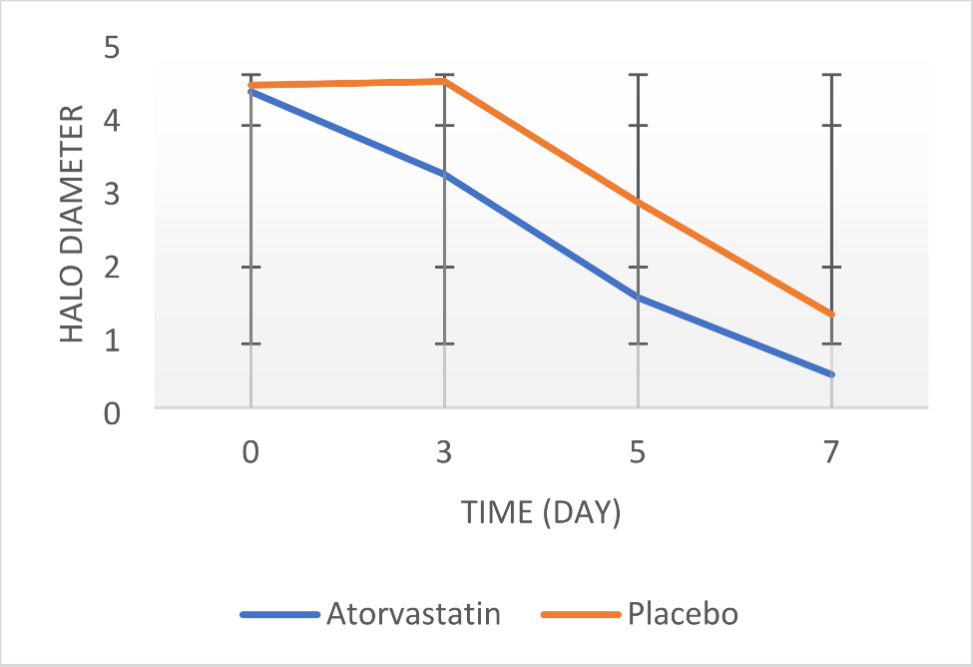
Halo diameter of treatment groups in time intervals

Based on Friedman’s test, an intra-group comparison of the results showed that halo diameter decreased significantly during the study period in both the intervention and control groups. Still, the intensity of the decrease was faster and more noticeable in the atorvastatin group (P = 0.000) (Table 1) (Fig. 2). Inter-group comparison of halo diameter shows that there is no statistically significant difference between the two groups on day zero of the study (P = 0.826). Still, on the third, fifth, and seventh days of the study, a significant difference was observed between the two groups, so that in the atorvastatin group, lesions decreased in size and improved more quickly (P < 0.05) (Fig. 2). Based on the results of the GEE test, the changes in the lesion diameter between the two groups were insignificant (P > 0.05).

**Table 1:** comparison of halo diameter and pain intensity during study in both groups

Variables	Days	Groups		Significance level (Independent T-test/ Mann-Whitney)	Significance level ( GEE*time )
		Atorvastatin	Placebo		
Halo diameter (mm)	0	1.32 ± 4.31	1.40 ± 4.40	0.826	0.072
	3	1.36 ± 3.18	1.26 ± 4.45	*0.003	
	5	1.18 ± 1.50	1.40 ± 2.8	∗0.003	
	7	0.59 ± 0.45	1.16 ± 1.27	∗0.014	
Significance level (Friedman)		0.000∗	0.000∗		
Pain intensity	1	1.37 ± 7.48	1.26 ± 7.45	0.936	0.162
	2	1.05 ± 6.31	1.51 ± 7.10	0.052	
	3	1.37 ± 4.84	1.57 ± 6.27	*0.003	
	4	1.33 ± 3.34	1.88 ± 5.28	*0.000	
	5	1.36 ± 2.63	1.85 ± 4.37	*0.001	
	6	1.01 ± 1.56	1.63 ± 2.78	*0.005	
	7	0.80 ± 0.98	1.30 ± 1.28	0.821	
Significance level (Friedman)		*0.000	*0.000		

The sign * indicates p-value < 0.05

### Pain intensity

**Fig. 3:**
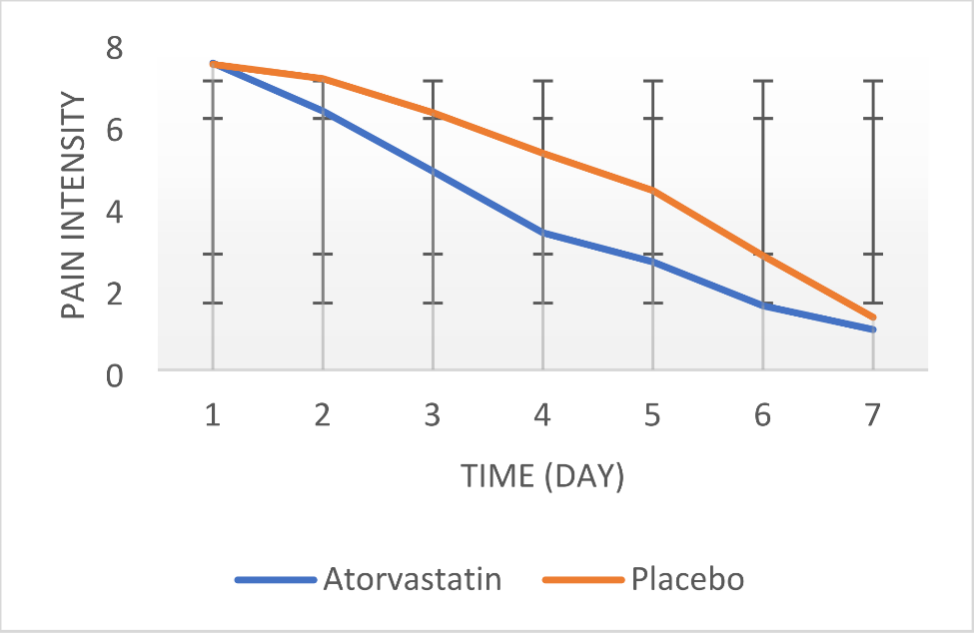
Pain intensity by treatment group in time intervals

Based on Friedman’s test results, intragroup comparison of patient pain intensity (VAS) showed a significant decrease from day one to seven in both intervention and control groups (P = 0.000) (Table 1) (Fig. 3).

In the inter-group comparison of pain intensity, no significant difference was found between the two groups on the first, second, and seventh days (P > 0.05). However, from the third day to the sixth day, the pain significantly decreased in the intervention group and the difference between the two groups was statistically significant (P < 0.05) (Table 1) (Fig. 3). Also, based on the results of the GEE test, there was no significant difference in the pain intensity scores between the two groups during the study period.

## Discussion

This study examined the effect of atorvastatin mucoadhesive tablets on the diameter of the inflammatory halo and the pain intensity of aphthous ulcers in patients. This study was conducted as a double-blinded clinical trial on 44 patients participating in the study (22 patients in the atorvastatin group and 22 patients in the placebo group), and the findings of this study were compared inter-groups and intra-groups. The results of this study show that the halo diameter in the atorvastatin group had significantly reduced compared to the placebo group, so that on the seventh day of the study, the aphthous lesions in the atorvastatin group had improved significantly. However, in the placebo group, the average diameter of the inflammatory halo was more than one millimeter. The pain intensity of participants (VAS) showed a notable decrease in the atorvastatin group except on days one, two, and seven of the study. In addition, the pain reduction process was faster in the atorvastatin group than in the control group.

Many studies have investigated the anti-inflammatory, analgesic, and antioxidant effects of atorvastatin [11, 18]. However, it is impossible to compare the results accurately due to the lack of a similar study on the effect of atorvastatin on minor aphthous lesions. Recurrent aphthous stomatitis is one of the most common oral diseases, characterized by the frequent occurrence of small oral lesions without any other symptoms. The etiology of this disease is multifactorial and inflammatory and is related to factors such as oxidative stress, lack of immune regulation, and lack of vitamins and minerals. Therefore, the suggested treatments for this condition are aimed at reducing symptoms and inflammation, as well as reducing the occurrence of lesions through reducing oxidative stress [19].

Statins are among the least complicated and most effective drugs used to treat blood lipid disorders. This category of drugs effectively reduces low-density lipoprotein in the blood; in addition, they have a beneficial outcomes in reducing oxidative stress, anti-inflammatory effects, immune modulation, analgesia, Etc. [20].

Most of the side effects mentioned for atorvastatin are related to anti-inflammatory properties because inflammation plays a significant role in the pathogenesis of various diseases such as cancer, arthritis, and Alzheimer’s. The anti-inflammatory function of these drugs is mainly related to the reduction of C-reactive protein, which is one of the most prominent factors in inflammation. However, in various studies, many other processes have been described for this function [18].

The analgesic effect of atorvastatin is caused by processes such as inhibiting cytokines, MMP-2, and NGF in the sciatic nerves and spinal cord, as well as increasing the level of antioxidants and reducing the production of prostaglandins, all of which act in peripheral sensory receptors [11]. Therefore, considering the anti-inflammatory, analgesic, and antioxidant properties of atorvastatin, which all play a role in the improvement of aphthous lesions, as well as the preference for local care in treating aphthous ulcers, we investigated the effect of atorvastatin mucoadhesive tablets on minor aphthous lesions in this study.

Masoumi et al. investigated the effect of oral atorvastatin and local injection of this drug on treating periodontitis in rats. This study states that both forms of atorvastatin reduce inflammation and the cascade of oxidant production and subsequent tissue destruction, as well as bone loss caused by collagen destruction in periodontitis. The results obtained from systemic use are not significantly different from topical use, but the topical form of this drug is preferable due to fewer side effects [21].

In a study, Tahamtan et al. systemically reviewed articles related to the effects of Statins on oral and dental health. In their research, due to the positive impact of Statins on bone metabolism, inflammation, and antioxidant properties, also strong effects on epithelialization and wound healing, and antibacterial, antiviral and, antifungal properties, the effect of this drug on oral health was investigated. The study’s results demonstrated that Statins have a significant impact on chronic periodontitis, implant osseointegration, orthodontic dental movements, soft and hard tissue repair, anticancer effects, Etc. [22].

Ghasias et al. investigated the antioxidant, analgesic, and anti-inflammatory function of atorvastatin and rosuvastatin in different animal models, the findings showed that these two medicines have no effect on central pain receptors, but their analgesic property is due to their role in peripheral analgesic processes so that they observed a remarkable inhibition of pain caused by acetic acid, which leads to pain sensation by increasing the levels of PGE2 and PGF2a. It was also observed that atorvastatin reduces pain sensitivity by reducing bradykinin and cytokines; as a result, the analgesic effect observed in atorvastatin is similar to the effect of anti-inflammatory agents. Examining the anti-inflammatory effect of these two medicines on acute and chronic inflammation showed that atorvastatin reduces acute inflammation by inhibiting the release of bradykinin, prostaglandin, and substance P. In chronic inflammation, these medicines reduce the mass of granuloma by inhibiting the proliferative phase of inflammation. The oxidative stress reduction action of these two drugs is also due to the increase in cellular antioxidants, the decrease in NADPH oxidase expression, and the regulation of catalase overexpression [18].

Nowadays, the use of mucoadhesive tablets made with herbals and chemicals is a common method in the treatment of aphthous lesions, and many studies have been conducted in this field, which includes the efficacy of mucoadhesive tablets containing propolis, licorice, and zinc sulfate on aphthous lesions. The results of these studies were favorable and promising because the combination of the medicines with mucoadhesive tablets has anti-inflammatory properties; on the other hand, the tablets last longer at the lesion site and increase the treatment effectiveness [23–25].

In general, the use of topical mucoadhesive tablets is a new and efficient method against mechanical trauma and inflammation of the lesion. In fact, in this method, a protective layer is created on the lesion as an auxiliary method and decreases the pain and discomfort caused by aphthous lesions and eliminates the lesion faster. Among the other advantages of this method, we can indicate the prevention of secondary infection of the ulcer, and as a result, no antibiotic or antifungal prescription will be needed [26].

According to the previous studies and considering the findings of the present study, it seems that the significant reduction in the size of the aphthous lesion and the pain intensity of patients in the atorvastatin group compared with a control group is related to the anti-inflammatory, analgesic or antioxidant properties of atorvastatin. However, due to the lack of similar articles in this field, we cannot only rely on this study and judge atorvastatin mucoadhesive tablets efficacy, and more studies are needed on this subject. In addition, the lack of direct access to patients during the seven days of study can be considered another limitation of this study.

## Conclusion

The results of this study show that the atorvastatin mucoadhesive tablets effectively reduce the pain and discomfort of patients and cause the healing and size reduction of aphthous lesions. So these tablets may be effective in the treatment of patients with recurrent aphthous stomatitis due to their anti-inflammatory and wound-healing properties. However, further studies with larger sample sizes should be conducted. Furthermore, the effect of these tablets on the recurrence of RAS and the application of these tablets in treating other oral lesions can be considered in future studies.

## Strength and limitations

This is the first study that examined the effect of atorvastatin mucoadhesive tablets on recurrent aphthous stomatitis. Therefore, comparing of this study with the results of similar studies was not possible due to the novelty of this research. More studies in this field with a larger sample size are required for more reliable findings. It is also notable that the lack of access to participants during the study was another limitation of this study, and controlling the patients’ diet as a factor influencing the study results was impossible.

## Data Availability

All data generated or analyzed during this study are included in this published article.

## Abbreviations

**VAS**: Visual Analogue Scale
**RAS**: Recurrent Aphthous Stomatitis
**GEE**: Generalized Estimated Equations
